# Epigenetic mechanisms underlying cognitive impairment and Alzheimer disease hallmarks in 5XFAD mice

**DOI:** 10.18632/aging.100906

**Published:** 2016-03-21

**Authors:** Christian Griñán-Ferré, Sara Sarroca, Aleksandra Ivanova, Dolors Puigoriol-Illamola, Fernando Aguado, Antoni Camins, Coral Sanfeliu, Mercè Pallàs

**Affiliations:** ^1^ Department of Pharmacology and Therapeutic Chemistry (Pharmacology Section) and Institute of Neuroscience, University of Barcelona, 08028 Barcelona, Spain; ^2^ Department of Cellular Biology, University of Barcelona, 08028 Barcelona, Spain; ^3^ Institut d'Investigacions Biomèdiques de Barcelona (IIBB), CSIC, and IDIBAPS, 08036 Barcelona, Spain

**Keywords:** Alzheimer disease, neurodegeneration, behavior, cognition, deacetylase, methyltransferase

## Abstract

5XFAD is an early-onset mouse transgenic model of Alzheimer disease (AD). Up to now there are no studies that focus on the epigenetic changes produced as a result of Aβ-42 accumulation and the possible involvement in the different expression of related AD-genes. Under several behavioral and cognition test, we found impairment in memory and psychoemotional changes in female 5XFAD mice in reference to wild type that worsens with age.

Cognitive changes correlated with alterations on protein level analysis and gene expression of markers related with tau aberrant phosphorylation, amyloidogenic pathway (*APP, BACE1*), Oxidative Stress (*iNOS, Aldh2*) and inflammation (astrogliosis, *TNF-α* and *IL-6*); no changes were found in non-amyloidogenic pathway indicators such as ADAM10.

Epigenetics changes as higher CpG methylation and transcriptional changes in DNA methyltransferases (DNMTs) family were found. *Dnmt1* increases in younger 5XFAD and *Dnmt3a* and *b* high levels in the oldest transgenic mice. Similar pattern was found with histone methyltransferases such as *Jarid1a* and *G9a*. Histone deacetylase 2 (*Hdac2*) or *Sirt6*., both related with cognition and memory, presented a similar pattern. Taken together, these hallmarks presented by the 5XFAD model prompted its use in assessing different potential therapeutic interventions based on epigenetic targets after earlier amyloid deposition.

## INTRODUCTION

Alzheimer's disease (AD) is a progressive and irreversible neurodegenerative disorder [1] and the most common cause of dementia, affecting about 35 million people worldwide [2]. A three-fold increase there is estimated for the number of cases of AD (114 million) by 2050 [1]. According to statistical analyses approved by the World Health Organization, (WHO), AD and the others forms of dementia rank as the fourth cause of death in economically developed countries with a rate of 4,1% of total deaths [3,4]. The disease involves degeneration of certain regions of the brain, which results in memory loss and declining cognitive functions and leads to a decrease in physiological functions and death. The neuropathological hallmarks comprise the production of senile plaques, the formation of neurofibrillary tangles, and the accumulation of neuronal lesions in the brain [5]. Despite that Late-Onset AD (LOAD) has a relatively high heritability of around 70% [6], the sole long-established, unequivocal genetic risk factor has been the ε4-allele of the Apolipoprotein E gene (*APOE*) [7-10]. The precise mechanism by which *APOE* exerts this influence remains largely undefined, with indications including a key role in mediating Aβ metabolism and clearance [11]. Numerous candidate gene, genetic linkage, and association studies have been carried out over the past two decades to elucidate the remaining genetic risk for AD [12]. However, none of these candidates has proven to consistently influence disease risk or onset age in more than a handful of samples [13]. Reasons for lack of reproducibility may include the following: insufficient study power to detect variants with minor contributions; biologic, genetic, and allelic heterogeneity; difference in study design, and the presence of population substructure [14]. The occurrence of phenotypical differences in monozygotic twins over time is thought to arise from epigenetic changes induced by different environments or by stochastic events [15]; thus, it appears plausible that epigenetic changes resulting in altered gene expression may also be involved in the pathogenesis of LOAD.

In addition to genetic factors, growing evidences demonstrated that the effects of environmental factors and epigenetic mechanisms also play an important role in the pathogenesis of AD [16-20]. Epigenetic information is principally encoded in two types of synergistically acting covalent modifications: DNA methylation and chromatin modifications; these changes afford the epigenetic complexity that directly affects regulation of the gene expression and other genomic functions [21]. For example, hypomethylation of the Amyloid Precursor Protein (APP) gene in the brain of a patient with AD was found [22]. Subsequently, several studies have linked DNA methylation changes in hippocampus and cortex of patients with AD, showing that DNA hypomethylation is associated with a huge number of Amyloid β (Aβ) plaques [22]. DNA methylation is also involved in APP processing and Aβ production through the regulation of Presenilin1 (PS1) and β-secretase (BACE) expression [23]. Monitoring the frontal cortex of patients with AD, indicated that DNA hypomethylation leads to expanded regulation of proinflammatory genes, Nuclear Factor-κβ (NF-κβ) and the encoding of cyclooxygenase-2 that catalyzes the production of prostaglandins and some prostanoids [24,25]. This assumes that abnormal DNA methylation may cause nerve inflammation. Hypermethylation of Brain Derived Neurotrophic Factor (BDNF) and cAMP Response Element Binding (CREB) promoters would harm the plasticity of the synapses [25]. Although these studies report AD brain global DNA methylation changes in opposite directions, differences are likely due to the fact that the authors studied different brain regions and employed different methylation detection techniques.

In addition, Aβ changes in DNA methylation are associated with induction of several genes, causing apoptotic and cell loss. It remains unclear how these changes in DNA methylation began, but it is suggested that various cellular mechanisms, such as OS and Aβ-42, are involved [26]. OS and Aβ-42 may modify DNA methylation, and evidences for the dynamic modulation of learning and memory suggest the functional importance of abnormal methylation of DNA in the cases of AD [27].

The detection and identification of mutations in APP, PS1 and PS2 genes associated with Familial AD (FAD) have allowed the generation of various transgenic murine models. These transgenic models have the potential to enable us to understand the pathogenesis of the disease, and to develop and evaluate new and effective therapeutic targets. The 5XFAD mouse was formally established and declared in 2006 by Oakley and the specific characteristic of this model being that it is generated to promote rapid, aggressive and complete development of AD pathological interactions. This novel model of Aβ pathology consists of two APP mutations (APP K67ON/M671L (Swedish), I716V (Florida), and V717 (London) and two PS1 mutations (PS1 M146 and L286V) on B6/SJL and each transgene is regulated by Thy1 promoter [28,29].

The present work focuses on the study of the possible involvement of Aβ-42 accumulation in the production of OS and in subsequent modifications in the epigenetic machinery in the murine model of AD, 5XFAD. We aimed to discuss the role of global DNA methylation and the histone epigenetic enzymes involved in epigenetic modification acting in the control of gene expression and memory in this AD mouse model.

## RESULTS

### 5XFAD showed cognitive impairment but no changes in emotional parameters with age

**Figure 1:**
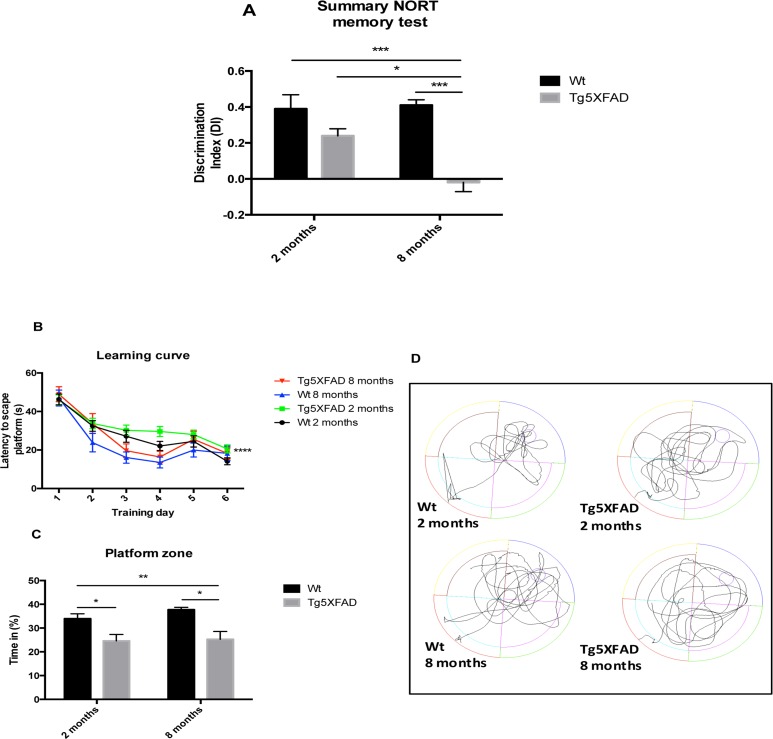
Results of Discrimination index of NORT in female mice aged 2 and 8 months (Wt and 5XFAD) (**A**). Learning curves during MWM trials (**B**), Percentage of time spent in platform zone during 60 sec probe trial of the MWM test (**C**), Representative swim paths from the memory day test on day 7 (**D**). Data represented as observed mean ± Standard Error of the Mean (SEM) (*n* = 10 for each group). *p<0.05; **p<0.01; ***p<0.001.

NORT analysis demonstrated significant differences between the 5XFAD and Wt strains at 8, but not at 2 months of age (age, F(1,28) = 4.5631, p = 0.0416; genotype, F(1,28) = 4.563, p = 0.001, and interaction age × genotype F(1,28) = 6,211, p = 0.0189). The DI was significantly decreased in 5XFAD mice at 8 months of age compared with young 5XFAD mice and with age-matched Wt, indicating impairment in hippocampal memory processes (Fig. 1A). Although, all mouse groups were able to learn through trial days in the MWM paradigm, statistical differences were found comparing young 5XFAD compared with old Wt. (Fig. 1B). On the test day, it was observed that 5XFAD mice aged 2 and 8 months showed impairment in spatial memory, with a significant decrease in time spent in the platform quadrant and with erratic preference for the platform area (age, F (1,28) = 0.8253, p = 0.3720; genotype, F(1,28) = 19.67, p = 0.0001, and interaction age × genotype F(1,28) = 0.3977, p = 0.5334) (Figs. 1C and 1D).

**Figure 2:**
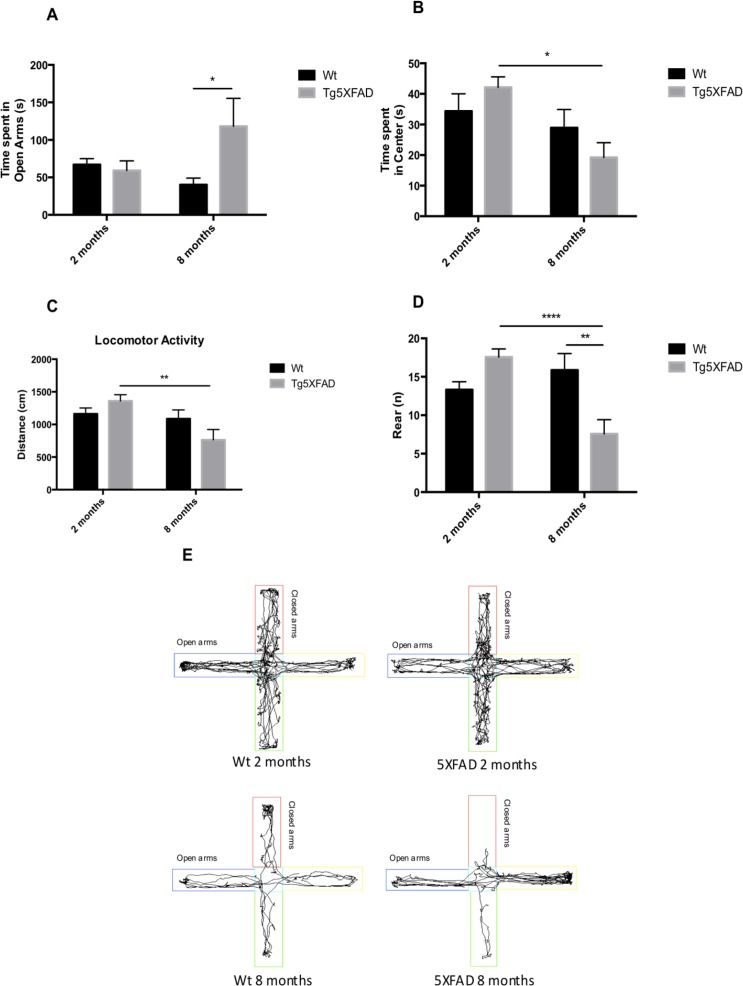
Results of Elevated Plus Maze (EPM) in female mice aged 2 and 8 months (Wt and 5XFAD). Time spent in open arms (**A**), Time spent in center maze (**B**), Locomotor activity (**C**), Rears/vertical activity (**D**), Representative track sheets showing duration dependent changes in time spent in and number of entries on the open arm and closed arm in EPM (**E**). Data represented as observed mean ± Standard Error of the Mean (SEM); (*n* = 10 for each group). *p<0.05; **p<0.01; ****p<0.0001.

**Figure 3:**
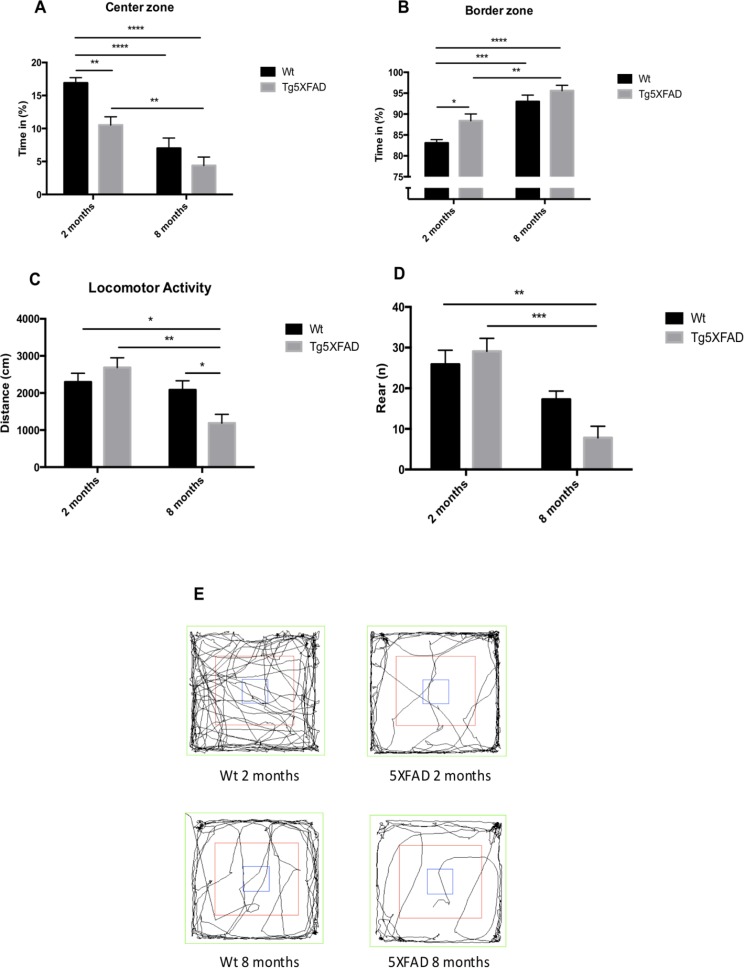
Results of Open field test in female mice aged 2 and 8 months (Wt and 5XFAD). Time spent in center zone., (**A**), Time spent in border maze (**B**), Locomotor activity (**C**) and Rears (**D**). Representative tracks for the OFT (**E**). Data represented as observed mean ± Standard Error of the Mean (SEM) (*n* = 10 for each group). *p<0.05; **p<0.01; ***p<0.001; ****p<0.0001.

Behavioral and emotional tests showed differences in anxiety-like behavior, through EPM test (Figs. 2A and 2B). Old 5XFAD mice demonstrated less anxious behavior, spending a longer time in open that in closed arms (age, F(1,32) = 0.7973, n.s.; genotype, F(1,32) = 3.842, p = 0.0587, and interaction age × genotype F(1,32) = 5.761, p = 0.0024). Locomotor activity was reduced significantly in 5XFAD (age, F(1,32) = 8.123, p = 0.0076; genotype, F(1,32) = 0.2784, n.s., and interaction age × genotype F(1,32) = 6.921, p = 0.0338) (Fig. 2C and E) whereas vertical activity in EPM was significantly reduced in old 5XFAD (age, F(1,32) = 6.167, p <0.0184; genotype, F(1,32) = 1.827, n.s., and interaction age × genotype F(1,28) = 6,211, p = 0.0189) (Fig. 2D). A summary of the parameters measured are presented in Table 2. O The OFT demonstrated that both female Wt and 5XFAD exhibited increased fear with age, reducing time in center zone (age, F(1,28) = 43.25, p < 0.0001; genotype, F(1,28) = 13.57, p = 0.001, and interaction age × genotype F(1,28) = 2.887, n.s.) (Fig. 3A), increasing time in border zone (age, F(1,28) = 34.69, p < 0.001; genotype, F(1,28) = 8.494, p = 0.0069, and interaction age × genotype F(1,28) = 0.9871, n.s.) (Fig. 3B). According to the results obtained in EPM paradigm, locomotor activity was reduced in 5XFAD aged 8 months (age, F(1,28) = 11.73, p =0.0019; geno-type, F(1,28) = 1.021, n.s., and interaction age × geno-type F(1,28) = 6,625, p = 0.0156) and vertical activity or rears (age, F(1,28) = 23.33, p < 0.0001; genotype, F(1,28) = 1.029, n.s., and interaction age × genotype F(1,28) = 4.190, p = 0.0502) (Figs. 3C-E). A summary of results obtained in the OFT is depicted in Table 3.

**Table 1:** Antibodies used in Western blot and Inmunohistochemical studies

Antibody	Host	Source/Catalog	WB dilution	ICH dilution
**BACE1**	Rabbit	Abcam/ab5832	1:500	
**4-HNE**	Rabbit	Abcam/ab46545	1:1000	
**SOD1**	Sheep	Calbiochem/574597	1:1000	
**sAPP beta**	Rabbit	Covance/SIG-39138-050	1:1000	
**Sinaptophysin**	Mouse	Dako/Clone SY38	1:2000	
**GAPDH**	Mouse	Millipore/MAB374	1:2000	
**NeuN**	Mouse	Millipore/MAB377	1:1000	
**GFAP**	Mouse	Abcam/ab48050-100		1:1000
**TAU s396**	Rabbit	Invitrogen/44752G	1:1000	
**TAU total**	Goat	Santa cruz/sc-1995	1:1000	
**Alexa Fluor 546 donkey anti-rabbit IgG A**		Molecular probes/AF488:A21202		1:400
**Alexa Fluor 488 donkey anti-mouse IgG A Alexa**		Molecular probes/AF555:A31572		1:400
**Donkey-anti-goat HRP conjugated**		Santa Cruz Biotech/sc-2020	1:3000	
**Goat-anti-mouse HRP conjugated**		Biorad/# 170-5047	1:2000	
**Goat-anti-rabbit HRP conjugated**		Cell Signaling/# 7074	1:2000	

**Table 2:** Parameters measured in the Elevated Plus Maze Test (EPM). (n): number of events

	Wt 2 months	5XFAD2 months	Wt 8 months	5XFAD8 months
**Locomotor activity (cm)**	1,161.79 ± 89.91	1,360.81 ± 94.74	1,087.42 ± 135.49	764.18 ± 157.50^##^
**Time in zone-Center (sec)**	34.35 ± 5.67	42.13 ± 3.45	28.93 ± 6.42	19.22 ± 4.86^#^
**Time in zone- Open Arms (sec)**	67.16 ± 7.93	59.28 ± 12.85	40.17 ± 8.96	118.25 ± 37.15^$^
**Time in zone- Closed Arms (sec)**	198.11 ± 10.03	198.06 ± 14.87	230.15 ± 13.43	162.23 ± 35.18
**Rearings (n)**	13.33 ± 1.02	17.58 ± 1.05	15.88 ± 2.15	7.57 ± 1.86^####^,^$$^
**Defecations (n)**	0.67 ± 0.27	0.83 ± 0.33	0.75 ± 0.49	0.29 ± 0.29
**Urinations (n)**	0.22 ± 0.17	0.17 ± 0.14	0.25 ± 0.16	0.14 ± 0.14

Results are expressed as a mean ± Standard error of the mean (SEM).

* *p* <0.05

** *p* <0.05

*** *p*<0.001 vs Wt, 2 months

# *p* <0.05

## *p* <0.001 vs 5XFAD, 2 months

$ p<0.05 vs Wt, 8 months.

**Table 3:** Parameters measured in the Open Field Test (OFT)

	Wt 2 months	5XFAD2 months	Wt 8 months	5XFAD8 months
**Total Distance (cm)**	2,296.28 ± 235.08	2,686.37 ± 260.57	2,083 ± 246.02	1,383.31 ± 291.40*,^##^,^$^
**Distance in Zone Center (cm)**	46.64 ± 10.01	40.62 ± 8.06**	266.05 ± 48.34****	166.72 ± 44.46****,^##^
**Distance in Zone Periphery**	2,249.65 ± 228.66	2,645.76 ± 258.96*	1,817.83 ± 221.38***	1,218.98 ± 255.46****,^##^
**Center (%)**	16.91 ± 1.82	11.60 ± 1.63	7.00 ± 1.55**	4.81 ± 1.26***,^#^
**Periphery (%)**	83.09 ± 0.80	88.40 ± 1.63*	93.00 ± 1.55****	95.19 ± 1.26****,^##^
**Rearings (n)**	25.89 ± 3.45	29.08 ± 3.20	17.29 ± 2.04	7.29 ± 2.68**,^###^
**Defecations (n)**	0.44 ± 0.19	0.33 ± 0.25	1.14 ± 0.51	0.57 ± 0.20
**Urinations (n)**	0.33 ± 0.18	0.08 ± 0.11	0.00 ± 0.00	0.00 ± 0.00

(n): number of events. Results are expressed as a mean ± Standard error of the mean (SEM)

* *p* <0.05

** *p* <0.05

*** *p*<0.001 vs Wt, 2 months

# *p* <0.05

## *p* <0.001 vs 5XFAD, 2 months

$ p<0.05 vs Wt, 8 months.

### Alzheimer's disease hallmarks and oxidative stress in female 5XFAD: changes with age

**Figure 4:**
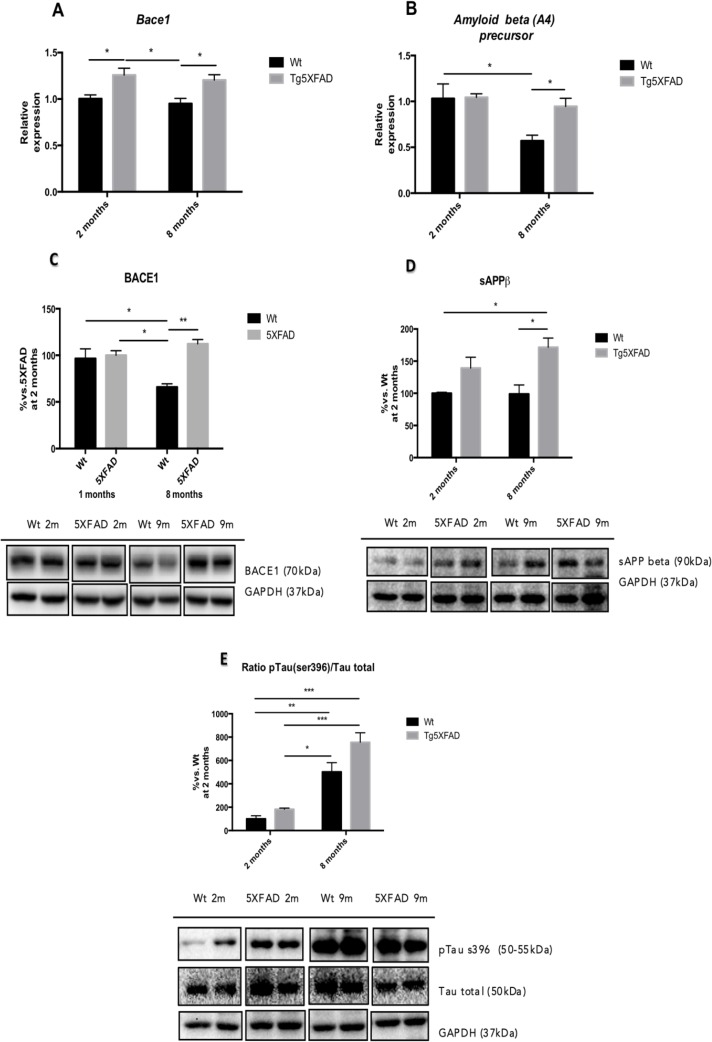
β-amyloid pathway gene expression in in female mice aged 2 and 8 months (Wt and 5XFAD) for Bace1 (**A**), *Amyloid Beta (A4) precursor* (**B**) and representative Western blot for BACE1 (**C**), sAPP (**D**) and p-Tau (Ser 396) /Tau ratio quantifications (**E**). Bars represent mean ± Standard Error of the Mean (SEM), and values are adjusted to 100% for levels Wt at 2 months. **p<0.05; **p<0.01; ***p<0.001.

Molecular analysis demonstrated that there a significant increase in BACE1 and APP gene expression (Figs. 4A and 4B) in 8 month-old 5XFAD compared with Wt (BACE1:age, F(1,8) = 0.8420, n.s.; genotype, F(1,8) = 19.13, p = 0.0009, and interaction age × genotype F(1,8) = 0.0004, n.s., and APP: a ge, F(1,8) = 8.292, p = 0.0138; genotype, F(1,8) = 3.969, n.s., and interaction age × genotype F(1,8) = 3.475; n.s.).

According to gene expression results, when protein levels for BACE1 were studied significant increases of BACE1 in 5XFAD in comparison with Wt were demonstrated (age, F(1,8) = 0.5599, n.s.; genotype, F(1,8) = 15.05, p = 0.0047, and interaction age × genotype F(1,8) = 11.17, p = 0.0102) (Fig. 4C). sAPPβ protein levels were studied as a indicative of APP processing and Aβ production; the results showed that sAPPβ levels increased in 8 month-old 5XFAD compared with age-mated Wt (age, F(1,8) = 1.647, n.s.; genotype, F(1,8) = 1.449, n.s., and interaction age × genotype F(1,8) = 18.45, p = 0.0026 (Fig. 4D). This is in agreement with increases in thioflavine-S staining in 5XFAD in front over Wt, as described elsewhere (age, F(1,8) = 149.6, p<0.0001.; genotype, F(1,8) = 278.2, p<0.0001., and interaction age × genotype F(1,8) = 149.6, p <0.0001) (Figs. 7A and 7B).

Tau hyperphosphorylation was also studied with a focus on pTau (Ser396), whose levels exhibited an age dependent increase in pTau both in Wt and in 5XFAD mice (age, F(1,8) = 69.06, p <0.0001; genotype, F(1,8) = 8.256, p = 0.0207, and interaction age × genotype F(1,8) = 2.108, n.s.) (Fig. 4E).

**Figure 5:**
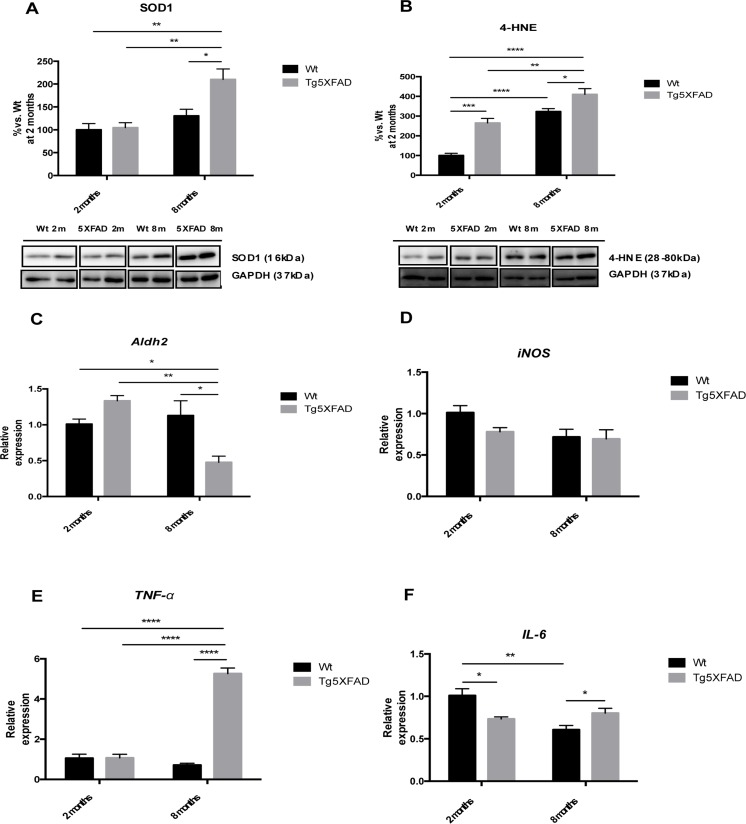
Oxidative stress and pro-inflammatory markers in female mice aged 2 and 8 months (Wt and 5XFAD), representative Western blot for SOD1 (**A**), 4-HNE (**B**), representative gene expression for *Aldh2* (**C**), *iNOS* (**D**), *TNF-α* (**E**), and *IL-6* (**F**). Mean ± Standard Error of the Mean (SEM) from five independent experiments performed in triplicate are represented. *p<0.05; **p<0.01; ***p<0.001; ****p<0.0001.

OS is an earlier event in AD mouse models, including 5XFAD. Increased levels in SOD1 (age F(1,8) = 17.9969, p <0.0028; genotype, F(1,8) = 6.875, p = 0.0306, and interaction age × genotype F(1,8) = 5.446, p = 0.049), jointly with 4-HNE (age, F(1,8) = 76.55, p <0.0001; genotype, F(1,8) = 36.13 p = 0.0003, and interaction age × genotype F(1,8) = 3.451, n.s.) (Figs. 5A and 5B) were found in 5XFAD older mice and in reference to Wt. According to an increase in 4-HNE diminutions in *Aldh2* enzyme transcription levels were demonstrated in older 5XFAD (age, F(1,8) = 9.098, p =0.0107; genotype, F(1,8) = 1.789, p = n.s., and interaction age × genotype F(1,8) = 16.06, p = 0.0017) (Fig. 5C) although not differences were found for *iNOS* gene expression among the studied groups (Fig. 5D). With reference to inflammation development, significant gene upregulation of *TNF-α* (age, F(1,12) = 97.05, p <0.001; genotype, F(1,12) = 136.4, p < 0.0001, and interaction age × genotype F(1,12) = 135.0, p < 0.0001) and *IL-6* (age, F(1,12) = 17.74, p <0.0119; genotype, F(1,12) = 51.40, p < 0.04871, and interaction age × genotype F(1,12) = 17.74, p < 0.0012) were found among animal groups. Both cytokines were significant higher in 8 months-old 5XFAD mice compared with young animals and age mated Wt indicating an age dependent inflammatory process (Figs. 5C and 5D).

**Figure 6:**
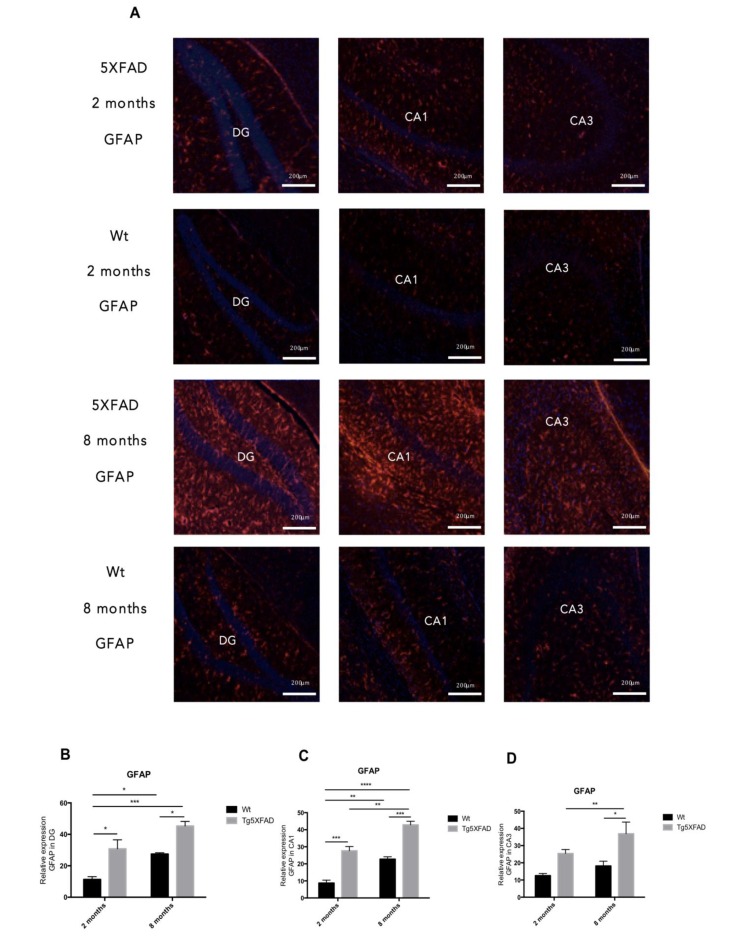
Representative images for GFAP immunostaining (**A**) and quantification on the bar chart (**B-D**) in female mice aged 2 and 8 months (Wt and 5XFAD). Bars represent mean ± Standard Error of the Mean (SEM); (*n* = 4 for each group). ***p<0.001; ****p<0.0001. DG: Dentate Gyrus. Scale bar for immunohistochemical images is 200 μm.

OS leads to gliosis and neuronal loss. In our hands increases in Glial Fibrillary Acidic Protein (GFAP) staining was found in 5XFAD animals compared with Wt, indicating harmful environment (Fig. 6A).

**Figure 7:**
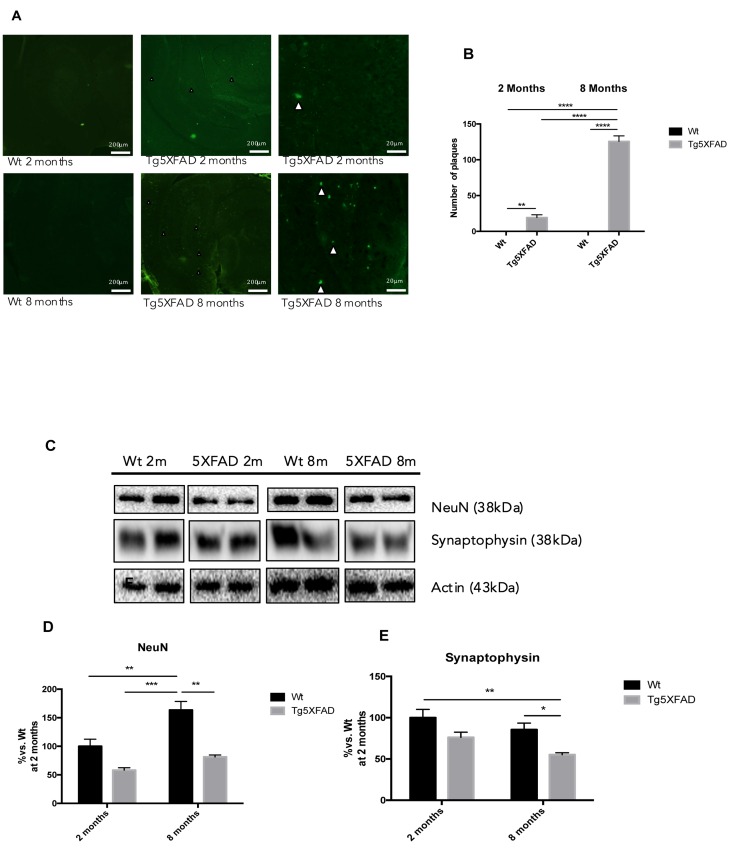
Histological images of amyloid plaques stained with thioflavin-S in female mice aged 2 and 8 months (Wt and 5XFAD). There is a heavy load of plaques in the majority of the brain areas ilustrated in 5XFAD the brain section (**A**). Representative Western blot for NeuN **(B, C)** and Synaptophysin (**D, E**). Bars represent mean ± Standard Error of the Mean (SEM), *n* = 4 for each group; DG: Dentate Gyrus. Scale bar for histochemical images is indicated in the Picture; *p<0.05; **p<0.01; ***p<0.001.

Quantification of fluorescence intensity demonstrated higher astrogliosis in hippocampus of 5XFAD with regard to Wt and significant worsening with age (See Figs. 6B-D for statistical notations). Moreover, significant neuronal loss measured as NeuN levels accompanied gliosis with strain and age (Figs. 7C and 7D) (age, F(1,8) = 4.029, p = 0.0796; genotype, F(1,8) = 18.53, p = 0.0026, and interaction age × genotype F(1,8) = 4.029, n.s.). On the other hand, reduction in synaptic structures, measured by Synaptophysin protein levels was also determined in 5XFAD in reference to Wt mice (Figs. 7C-E) (age, F(1,8) = 6.047, n.s.; genotype, F(1,8) = 14.14, p = 0.0055, and interaction age × genotype F(1,8) = 0.211, n.s.).

### Epigenetic enzymes gene expression differences in female 5XFAD

**Figure 8:**
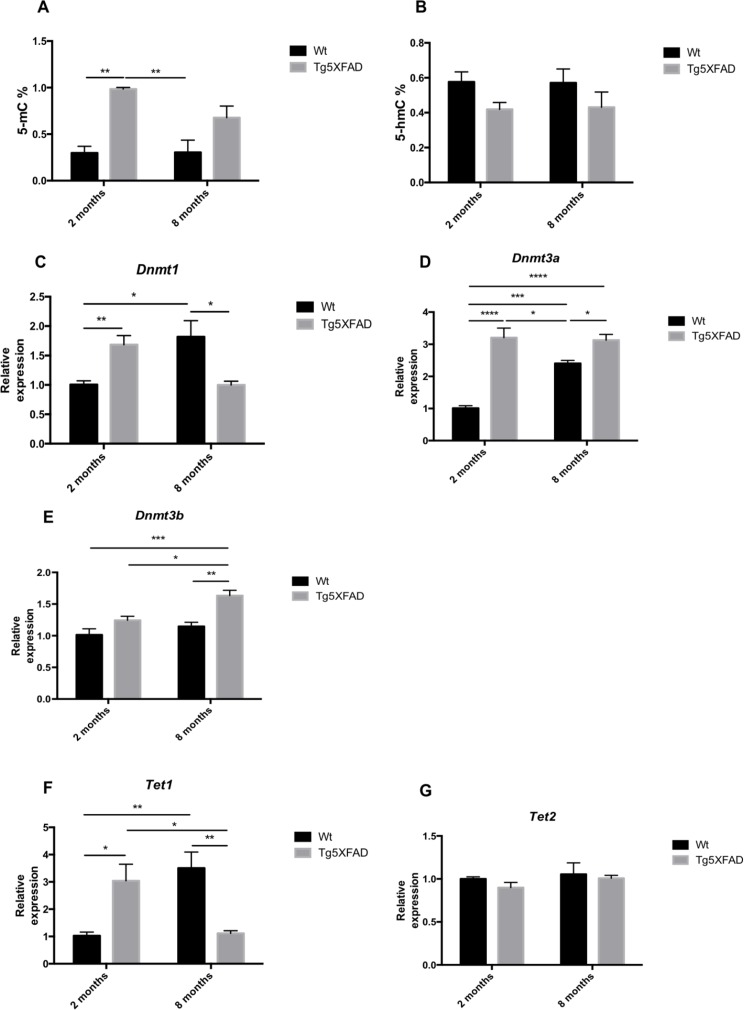
Global 5-methylated cytosine (**A**) and 5-hydroxymethylated cytosine levels (**B**) in female mice aged 2 and 8 months (Wt and 5XFAD). Gene expression for *Dnmt1* (**C**)*, Dnmt3a* (**D**), *Dnmt3b* (**E**), *Tet1* (**F**), and *Tet 2* (**G**). Gene expression levels were determined by real-time PCR. Mean ± Standard Error of the Mean (SEM) from five independent experiments performed in triplicate are represented. *p<0.05; **p<0.01; ***p<0.001; ****p<0.0001.

Global methylation and hydroxymethylation was measured by ELISA. Results demostrated a superior levels in DNA methylation (5-mC) in 5XFAD mice with respect to Wt which reached significance at the age of 2 (age, F(1,8) = 2.383, n.s.; genotype, F(1,8) = 29.32, p = 0.0006, and interaction age × genotype F(1,8) = 2.583, n.s.) (Fig. 8A), whereas 5-hmC presented a diminution trend, which was not statistically significant (Fig. 8B). DNMTs, HDMs and HDACs gene expression was determined by RT-PCR, and non-transgenic and 5XFAD mice at ages 2 and 8 months were compared. The results demonstrated a significant increase in the levels of *Dnmt1* in young transgenic mice, this rise is inverted at the older age being higher in Wt groups (age F(1,12) = 0.1417, n.s.; genotype, F(1,12) = 0.1785, n.s., and interaction age × genotype F(1,12) = 20.68, p = 0.007) (Fig 8C). *Dnmt3a* and *Dnmt3b* levels increased in the mice at 8 months of age, being significant higher in 5XFAD groups (*Dnmt3a*: age, F(1,12) = 12.90, p =0.0037; genotype, F(1,12) = 63.51, p < 0.0001, and interaction age × genotype F(1,12) = 16.18, p = 0.0017; *Dnmt3b*: age, F(1,12) = 11.06, p =0.0060; genotype, F(1,12) = 20.76, p < 0.0007, and interaction age × genotype F(1,12) = 2.668, n.s.) (Figs. 8D-E). TET oxidized 5-mC and create 5-hmC, which may represent epigenetic markers of their own with a role for priming the epigenome. *Tet1* was found significantly reduced in old 5XFAD in comparison with Wt (age, F(1,12) = 0.4151, n.s.; genotype, F(1,12) = 0.1934, n.s. and interaction age × genotype F(1,12) = 25.93, p = 0.003), without changes in *Tet2* (Fig. 8F).

**Figure 9:**
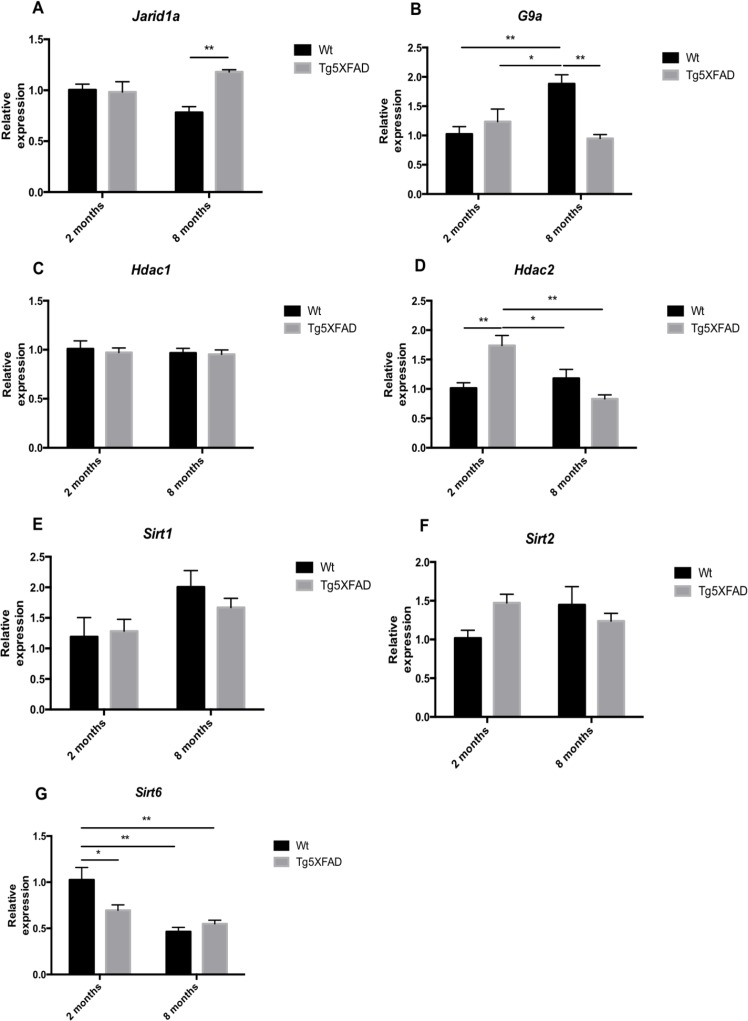
Gene expression for *Jarid1a* (**A**), *G9a* (**B**), *Hdac1* (**C**), *Hdac2* (**D**), *Sirt1* (**E**)*, Sirt2* (**F**), and *Sirt6* (**G**) in female mice aged 2 and 8 months (Wt and 5XFAD). Gene expression levels were determined by real-time PCR. Mean ± Standard Error of the Mean (SEM) from five independent experiments performed in triplicate are represented. *p<0.05; **p<0.01; ***p<0.001; ****p<0.0001.

Another epigenetic modification is histone demethylation and deacetylation. *Jarid1a*, a specific H3K4 histone demethylase, increased in older 5XFAD (age, F(1,12) = 12.90, p =0.0037; genotype, F(1,12) = 0.0367, n.s., and interaction age × genotype F(1,12) = 8.272, p = 0.0139) (Fig. 9A). In addition, the results demonstrated significant differences in the expression of *G9a* (a specific H3K9 histone methyltranferase) between non-transgenic and 5XFAD mice. In Wt mice, there was a significant G9a increase with age, that was absent in 5XFAD animals (age, F(1,12) = 3.604, n.s.; genotype, F(1,12) = 5.779, p = 0.0333, and interaction age × genotype F(1,12) = 14.61, p = 0.0024) (Fig. 9B). In terms of deacetylases, we observed a significant increase of *Hdac2* in 2-months-old 5XFAD in terms of Wt, but non-significant changes in older mice (age, F(1,12) = 8.394, p =0.0134; genotype, F(1,12) = 2.184, n.s., and interaction age × genotype F(1,12) = 17.60, p = 0.0012) (Fig. 9D). No differences in *Hdac1* gene expression of were found in 5XFAD mice compared with non-transgenic mice at any age (Fig. 9C). Sirtuin family deacetylases as with memory were also explored. No significant changes were found in the gene expression of *Sirt1* and *Sirt2* (Figs. 9E-F), but an earlier decrease in *Sirt6* expression was found in young transgenic mice, and in both strains in older mice (age, F(1,12) = 19.88, p =0.0008; genotype, F(1,12) = 2.378, n.s., and interaction age × genotype F(1,12) = 6.581, p = 0.0225) (Fig. 9G).

## DISCUSSION

Epigenetic modifications such as global DNA methylation/hydroxymethylation, histone demethylation levels and histone acetylation levels play a crucial role in gene regulation [30]. Epigenetic changes could be the basis for the presentation of senescence and for neurodegenerative diseases, such as AD [31]. In fact, impaired global DNA methylation has been found in aged and AD brain, but in addition other changes can be related with AD. For example, AD brain showed hyper- and hypomethylated CpG islands in promoter regions for CREB and NF-kB genes, respectively. Moreover, AD brain demonstrated increased global histone H3 acetylation and hypermethylation of the promotor region for the drebrin-like protein gene and many other epigenetic changes observed were inversely related with respective changes in messenger RNA and protein levels [25]. Because OS may be induced by Aβ, it can be an important contributor to DNA epigenetic modifications. It has been shown that ROS can command epigenetic modification by histone acetylation and deacetylation by HATs and HDACs, respectively, linking OS to chromatin remodeling [32]. However, the exact relationship among Aβ, ROS and epigenetics remains largely unknown, in terms of whether Aβ does, in fact, cause DNA methylation/demethylation or whether it is responsible for other epigenetic changes that exert an influence on the progression of AD.

5XFAD mice are characterized by higher levels in amyloidogenic pathology [33]. These mice accumulate high levels of intraneuronal Aβ-42 at around 1.5 months of age with amyloid deposition rapidly following at around two months of age, first in the subiculum and layer 5 of the cortex and increasing rapidly with age [34]. Plaques spread throughout the hippocampus and cortex by 6 months of age. Gliosis also begins at around 2 months of age, developing in parallel with plaque deposition. Synapse degeneration is also observed (at approximately 4 months of age) as well as neuronal loss and deficits in spatial learning (at approximately 4 to 5 months of age) [35]. Tangles are not typical in this model but tau hyperphosphorylation has been described [36,37]. We demonstrated, as expected, that in our hands female 5XFAD exhibited an increase in AD markers with age, including, an increase in *APP* and *Bace1* expression, higher protein levels of BACE1 and sAPPβ, and higher number of amyloid plaques indicating the development of AD pathology as described elsewhere in aged 5XFAD [38]. Another hallmark demonstrated in these transgenic animals comprised the increase in oxidative environment that parallels with the degree of disease development [39]. In our hands, oxidative stress initiated with 4-HNE increases at 2 months of age, but a complete development of oxidative markers was found at older ages, accordingly to the higher levels of amyloid pathology and also of tau hyperphosphorylation. AD brain undergoes a general OS process, as documented by significant protein oxidation, lipid peroxidation, and DNA oxidation [40]. Interestingly, 4-HNE, a reactive end-product of lipid peroxidation that is found increased in AD brains, has been observed to bind to histone lysine residues.

These molecular and biochemical findings are phenotypically demonstrated by loss in memory abilities and also by impairment in behavioral and emotional parameters in aged 5XFAD. Therefore, in our hands female 5XFAD initiate the AD characteristics as earlier as 2 months but the disease is completely developed at 8 months of age in a similar way described by Oakley and coworkers (2006).

Here we confront the implication of epigenetic changes with AD hallmarks in female 5XFAD (including Aβ deposition and OS processes), which we addressed from a holistic point of view, in order to demonstrate a possible relationship between the processes gated to earlier Aβ deposition that account for, in the 5XFAD brain, the epigenetic response, both to delay or to contribute the evolution of the disease.

By means of this approach, we demonstrate that a significant increase in global DNA methylation, measured by 5-methylCytosine (5-mC) levels in young animals when Aβ initiate deposition and with concomitant increase of *Dnmt1* and *Dnmt3a. Dnmt3b* is maintained as increased in older mice. Although no changes in hydroxymethylCytosine (5-hmC) levels were found, *Tet1* but not *Tet2* (which catalyze the conversion of 5-mC into 5-hmC) increases in 2 month-old transgenic mice, followed by a reduction in older animals. In reference to histone modification by acetylation or deacetylation, again we found differences in transgenic mice, with increases in *Hdac2, Jarid1a* or *G9a*.

It was described elsewhere that DNMT3a and DNMT3b are essential for memory formation and that they participated in neuronal and synaptic plasticity changes. Recently, it is has been reported that DNMT3b moderates cognitive decline in subjects with mild cognitive impairment [41,42]. The significant changes found in *Dnmt1* and *Dnmt3b* in young 5XFAD lead to higher cytosine methylation, prior to the development of AD hallmark in this mouse, underlying the earlier participation for methylation processed at the onset of the pathology. Some authors reported that in aged mice there occurs a diminution in hippocampal expression of *Dnmt3a*, and that increasing this expression reverses memory deficits whereas knockdown in young mice impairs memory formation [43]. The increases in *Dnmt3a* and *Dnmt3b* in transgenic animals could be an attempt to surpass the harmful effect of the Aβ increment as well as the high oxidant environment that occurs in 5XFAD when the disease is completely developed, as depicted in negative correlations found among the results obtained in the present work (Table 4).

**Table 4:** Partial correlation controlling for group coefficients between selected variables included in the study

Control Variables	NORTDI	EPM Time open arms	OFT Locomotor activity	Amyloidplaques	SOD1	4-HNE	DNMT1	HDAC2
NORT (DI)	1.000	−0.251	0.238	−0.485***	−0.155	0.241	0.344	0.469*
EPM Time open arms		1.000	−0.208	0.480***	0.327	0.330	−0.388	−0.392
OFT Locomotor activity			1.000	−0.424*	−0.308	−0.427*	0.096	0.368
Amyloid plaques				1.000	0.721***	0.005	0.633**	−0.750***
SOD1					1.000	0.442*	−0.361	−0.714***
4-HNE						1.000	0.402	−0.198
DNMT1							1.000	0.456*
HDAC2								1.000

The values used to calculate Partial correlation controlling for group coefficients were Amyloid pathology obtained with S-Thioflavin staining (showed in Figure 7B); behavioral parameters from NORT, EPM and OFT (showed in Figures 1A, 2A and 3C); protein expression for SOD1 and 4-HNE (showed in Figure 5A and 5B); and gene expression for *Dnmt1* and *Hdac2* (showed in Figures 8C and 9D) Correlation(2-tailed) is significant * p<0.05; **p<0.01 and ***p<0.001; (−) Negative covariation of two variables.

JumonjiC (JmjC) and ARID domain, containing histone lysine demethylase 1a (JARID1a) increased histone acetylation by inhibiting histone deacetylase 1 function and modifying gene transcription in a demethylase-independent manner. *Jarid1a* and *Jarid1b* catalyzed H3K4 demethylation contributing to the silencing of retinoblastoma target genes in senescent cells. Therefore Jarid1a and Jarid1b are tumor-suppressors that control cellular senescence [44]. Moreover, depletion of JARID1a in mammalian cells reduced per histone acetylation promoter, dampened gene expression and, for example, shortened the period of circadian rhythms [45].

Different histone methylations are also associated with behavioral disorders. G9a methylates H3K9 and this hypermethylation leads to the downregulation of BDNF [46]. It well established that BDNF plays an important role in neuron formation, maintenance and memory formation, and memory formation affects acetylation of H3 and H4 N-terminal tails. The lowest level of *G9a* gene expression in 5XFAD compared with non-transgenic mice is coincident with the loss of memory in this AD mouse model.

Finally, histone modification also plays an important role in epigenetic control. Histone deacetylases have been implicated in memory and cognition, and concretely, HDAC2 plays a central role in coupling lysine acetylation with synaptic plasticity and its modulation is implicated in cognition and disease [47]. Sirtuins are a family of nicotinamide adenine dinucleotide-dependent deacetylases that are implicated in a number of physiological and pathological processes, such as control of glucose and lipid metabolism, cancer, genomic stability and DNA repair [48]. SIRT6 plays a role in synaptic function and neuronal maturation and may be implicated in the regulation of neuronal survival [49] and the overexpression of the SIRT6 extended life span [50]. It is noteworthy that, in the 5XFAD model, *Hdac2* increased significantly in 2-month old transgenic mice whereas diminution of Sirt6 was found. No significant changes were found for these and other histone acetylases or deactetylases tested at older age (*Hdac1*, *Sirt1* and *Sirt2*). A very significant negative correlation between amyloid plaques and HDAC2 was found (Table 4), supporting the previously reported role of HDAC2 in synaptic plasticity [51] and working memory results obtained in 5XFAD.

Our results support that the Aβ pathology that is characteristic for 5XFAD mouse correlates with behavior, OS markers and specific epigenetic enzymes in 5XFAD (Table 4). Depending on disease stage development, DNA transcription modification processes, such as methylation or acetylation, may form part of a vicious cycle involving OS because the increase in Aβ accumulation, and that of, in turn, Aβ may induce global DNA changes in methylation and acetylation that correlated with the progression of the pathology from earlier onset to final stages of the disease in 5XFAD, including neuronal loss, gliosis and the disturbances in cognition and behavior present in this animal model of AD.

## METHODS

### Animals

Female Wild type (Wt, n = 10) and 5XFAD (Tg5XFAD, n = 10) mice at both 2 and 8 months of age, were used. These animals had *ad libitum* access to a standard chow diet (2018 Teklad Global 18% Protein Rodent Diet; Harlan Teklad, Madison, WI, USA) and tap water and were maintained under standard temperature conditions (22 ± 2°C) and 12h:12h light-dark cycles (300 lux/0 lux).

Studies were performed in accordance with the Institutional Guidelines for the Care and Use of Laboratory Animals established by the Ethical Committee for Animal Experimentation at the University of Barcelona.

### Behavioral and cognitive experiments Elevated Plus Maze test

This test is employed to assess anxiety [52]. The basic measurement is the animal's preference for dark, enclosed over bright, exposed places. The animal is placed in the center of the apparatus and observed for a set time. Measurements include total time spent in open and closed arms (and central platform), as well as entries into open and closed arms. Each mouse was individually placed at the center of the maze facing one of the enclosed arms and allowed to explore the maze freely during a 5-min observation period. Maze performance was video-recorded for later analysis. Time spent in open arms and numbers of arm entries were analyzed as indices of emotional behavior utilizing SMART^®^ ver. 3.0 software (PanLab, SLU, Spain).

### Open Field Test

Open Field Test (OFT) apparatus was constructed of white plywood (50 × 50 × 25 cm) [53]. Red lines were drawn on dividing the floor into 25-cm squares. Behavior was scored with SMART^®^ ver. 3.0 software, and each trial were recorded for later analysis, utilizing a camera fixed to the ceiling at a height of 2.1 m situated above the apparatus. Mice were placed at the center, or at one of the four corners, of the Open Field and allowed to explore the apparatus for 5 min. After the 5-min test, the mice were returned to their home cages and the Open Field was cleaned with 70% ethyl alcohol and allowed to dry between tests. To assess the animal's habituation process to the novelty of the arena, the mice were exposed to the apparatus for 5 min on 2 consecutive days. The behaviors scored included Line Crossing, Center Entries, Center Stay Duration, Rearing, Defecation, and Urination. Each animal was then given a score for total locomotor activity, which was calculated as the sum of total distance, line crosses and number of rears.

### Novel Object Recognition Test (NORT)

This test was conducted in a 90-degree, two-arm, 25-cm-long, 20-cm-high maze [54]. Light intensity in the middle of the field was 30 lux. The objects to be discriminated were plastic figures (object A, 5.25-cm high, and object B, 4.75-cm high). First, mice were individually habituated to the apparatus for 10 min for 3 days. On day 4, they were submitted to a 10-min acquisition trial (first trial), during which they were placed in the maze in the presence of two identical novel objects (A+A or B+B) placed at the end of each arm. A 10-min retention trial (second trial) occurred 2 h later. During this second trial, objects A and B were placed in the maze, and the times that the animal took to explore the new object (tn) and the old object (to) were recorded. A Discrimination Index (DI) was defined as (tn-to)/(tn+to). In order to avoid object preference biases, objects A and B were counterbalanced so that one half of the animals in each experimental group were first exposed to object A and then to object B, whereas the other one half first saw object B and then object A was presented. The maze, the surface, and the objects were cleaned with 96° ethanol between the animals' trials to eliminate olfactory cues.

### Morris Water Maze test

An open circular pool (100 cm in diameter, 50 cm in height) was filled halfway with water [55] and water temperature was maintained at 22°C ± 1. Two principal perpendicular axes were defined; thus, the water surface was divided into four quadrants (NE, SE, SW, and NW), and five starting points were set (NE, E, SE, S, and SW). Four visual clues were placed on the walls of the tank (N, E, S, and W). Non-toxic white latex paint was added to make the water opaque, and a white escape platform was submerged 1 cm below the water level (approximately in the middle of one of the quadrants).

The animal's swimming paths were recorded by a video camera mounted above the center of the pool, and the data were analyzed with SMART^®^ ver. 3.0 statistical software. The learning phase consisted of 6 days of trials for each mouse. The animals were submitted to five trials each day starting from the positions set (in random order) and without a resting phase between each trial and the subsequent one. At each trial, the mouse was placed gently into the water, facing the wall of the pool, and allowed to swim for 60 sec. If not able to locate the platform in this period of time, the mouse was guided to the platform by the investigator. Animals were left on the platform each time for 30 sec in order to allow spatial orientation.

The parameters measured were latency time in finding the platform, time spent in each quadrant, and distance swum for each trial; the mean was calculated for each trial day. A memory test was performed at the end of the learning days, in which the platform was removed and the time spent by each mouse in each quadrant was measured.

### Brain isolation and immunoanalysis assays

Mice were euthanized 3 days after the last trial was conducted, and the brain was quickly removed from the skull. Hippocampus were dissected and frozen in powdered dry ice and maintained at −80°C for further use. Tissue samples were homogenized in lysis buffer containing phosphatase and protease inhibitors (Protease Inhibitor Cocktail 2, Sigma), and cytosol and nuclear fractions was obtained as described elsewhere. Protein concentration was determined by the Bradford method. Fifteen μg of protein was separated by Sodium Dodecyl Sulfate-PolyAcrylamide Gel Electrophoresis (SDS-PAGE) (8(0002013)15%) and transferred onto PolyVinylidene DiFlouride (PVDF) membranes (Millipore). The membranes were blocked in 5% non-fat milk in Tris-Buffered Saline containing 0.1% Tween 20 (TBS-T) for 1 h at room temperature, followed by overnight incubation at 4°C with primary antibodies diluted in TBS-T and 5% Bovine Serum Albumin (BSA) (See details in Table 1). Membranes were then washed and incubated with secondary antibodies for 1 h at room temperature. Immunoreactive proteins were visualized utilizing an Enhanced ChemiLuminescence-based detection kit (ECL Kit; Millipore) and digital images were acquired employing a ChemiDoc XRS+System (BioRad). Band intensities were quantified by densitometric analysis utilizing Image Lab software (BioRad) and values were normalized to GAPDH.

For immunohistochemical studies, the frozen brains were embedded into OCT Cryostat Embedding Compound (Tissue-Tek, Torrance, CA, USA), cut into 20-μm-thick sections on a cryostat (Leyca Microsystems, Germany) at −20°C, and placed on slides. After 3 h of drying time at room temperature, the slices were fixed with acetone at 4°C for 10 min, allowed to dry overnight, and finally stored at (0002212)20°C until their further staining. For the staining procedure, the brain sections were first rehydrated by 5-min incubation in Phosphate-Buffered Solution (PBS). Afterward, the blocking/permeabilization step was performed (20 min in PBS 1% BSA + 1% Triton). Following two, 5-min washings in PBS, the slices were incubated overnight at 4°C with the primary antibodies (see Table 1 for dilutions). Two further washings were carried out prior to incubation with the fluorescent secondary antibody (1 h at room temperature, see Table for dilutions). Finally, before mounting with Fluoromount-G^™^ (EMS, Hatfield, NJ, USA), nuclear staining was performed with Hoechst 2 μg/mL for 5 min at room temperature. Slides were allowed to dry overnight after mounting and image acquisition was performed with a fluorescence laser microscope (Olympus BX41; Germany). At least four images from four different individuals by group were analyzed with ImageJ/Fiji software available online from the National Institutes of Health, NIH).

### Thioflavin S staining

Slides were allowed to defreeze at room temperature and then were rehydrated with PBS solution for 5 min. Later, the brain sections were incubated with 0.3% thioflavin S (Sigma-Aldrich) for 20 min at room temperature in the dark. Subsequently, these were submitted to washes in 3-min series, specifically with 80% ethanol (two washes), 90% ethanol (one wash), and three washes with PBS. Finally, the slides were mounted using Fluoromount (EMS), allowed to dry overnight at room temperature in the dark, and stored at 4°C. Image acquisition was performed with an epifluorescence microscope (BX41; Olympus, Germany). For plaque quantification, similar and comparable histological areas were selected, focusing on adjacent positioning of the hippocampus and the whole cortical area.

### RNA extraction and gene expression determination

Total RNA isolation was carried out by means of Trizol reagent following the manufacturer's instructions. RNA content in the samples was measured at 260 nm, and sample purity was determined by the A260/280 ratio in a NanoDrop™ ND-1000 (Thermo Scientific). Samples were also tested in an Agilent 2100B Bioanalyzer (Agilent Technologies) to determine the RNA integrity number. Reverse Transcription-Polymerase Chain Reaction (RT-PCR) was performed as follows: 2 μg of messenger RNA (mRNA) was reverse-transcribed using the High Capacity (complementary DNA) cDNA Reverse Transcription kit (Applied Biosystems). Real-time quantitative PCR (qPCR) was employed to quantify the mRNA expression of Aldehyde dehydrogenase 2 (*Aldh2*), amyloid beta A4 Precursor (*PreAPP*), β-secretase 1 (*Bace1*) and Disintegrin and Metalloproteinase 10 (*Adam10*), inflammatory genes Interleukin 6 (*IL-6*) and Tumor necrosis factor alpha (*TNF-α*), inducible Nitric Oxide Synthase (*iNOS*), epigenetic enzymes genes DNA (cytosine-5-)-methyltransferase 1 (*Dnmt1*), DNA (cytosine-5-)-methyltransferase 3 alpha (*Dnmt3a*), DNA (cytosine-5-)-methyltransferase 3 beta (*Dnmt3b*), Tet methylcytosine dioxygenase 1 (*Tet1*), Tet methylcytosine dioxygenase 2 (*Tet2*), lysine (K)-specific demethylase 5A (*Jarid1a*), histone-lysine N-methyltransferase 2 (*G9a*), histone deacetylase 1 (*Hdac1*), histone deacetylase 2 (*Hdac2*), sirtuin 1 (*Sirt1*), sirtuin 2 (*Sirt2*), sirtuin 6 (*Sirt6*). Normalization of expression levels was performed with *Actin* for SYBER Green and TATA-binding protein (*Tbp*) for TaqMan. The primers were as follows: for *Aldh2*, forward 5′-GCAGGCGTACACAGAAGTGA-3′ and reverse 5′-TGAGCTTCATCCCCTACCCA-3′, for *PreAPP*, forward 5′-AGGACTGACCACTCGACCAG-3′ and reverse 5′-CTTCCGAGATCTCTTCCGTCT-3′, for *Bace1*, forward 5′-AAGCTGCCGTCAAGTCCATC-3′ and reverse 5′-GCGGAAGGACTGATTGGTGA-3′, for *Adam10*, forward 5′-GGGAAGAAATGCAAGCTGAA-3′ and reverse 5′-CTGTACAGCAGGGTCCTTGAC-3′, for *IL-6*, forward 5′-ATCCAGTTGCCTTCTTGGGACTGA-3′ and reverse 5′-TAAGCCTCCGACTTGTGAAGTGGT-3′, for *TNF-α*, forward 5′-TCGGGGTGATCGGTCCCCAA-3′ and reverse 5′-TGGTTTGCTACGACGTGGGCT-3′, for *iNOS*, forward 5′-GGCAGCCTGTGAGACCTTTG-3′ and reverse 5′-GAAGCGTTTCGGGATCTGAA-3′, for *Dnmt1*, (Mm01151063_m1), for *Dnmt3a*, forward 5′-GGGCCACACGGCAGAG-3′ and reverse 5′-CACGGTTCTCCTCCTGTTCC-3′, for *Dnmt3b*, forward 5′-TGCCAGACCTTGGAAACCTC-3′ and reverse 5′-GCTGGCACCCTCTTCTTCAT-3′, for *Tet1*, forward 5′-CTGCCAACTACCCCAAACTCA-3′ and reverse 5′-TCGGGGTTTTGTCTTCCGTT-3′, for *Tet2*, forward 5′-CCATCATGTTGTGGGACGGA-3′ and reverse 5′-ATTCTGAGAACAGCGACGGT-3′, for *Jarid1a,* forward 5′-TCCGTGTGTCATCAGCCAAA-3′ and reverse 5′-CAAGCCTACGCCAGAGTCAA-3′, for *G9a*, forward 5′-TTCCTTGTCTCCCCTCCCAG-3′ and reverse 5′-GACGGTGACAGTGACAGAGG-3′, for *Hdac1,* forward 5′-TCACCGAATCCGCATGACTC-3′ and reverse 5′-TCTGGGCGAATAGAACGCAG-3′, for *Hdac2*, forward 5′-CTATCCCGCTCTGTGCCCT-3′ and reverse 5′-GAGGCTTCATGGGATGACCC-3′, for *Sirt1*, forward 5′-AACACACACACAAAATCCAGCA-3′ and reverse 5′-TGCAACCTGCTCCAAGGTAT-3′, for *Sirt2*, forward 5′-TGCAGGAGGCTCAGG ATTC-3′ and reverse 5′-GTCACTCCTTCGAGGGTCAG-3′, for *Sirt6*, forward 5′-GTCTCACTGTGTCCCTTGTCC-3′ and reverse 5′-GCGGGTGTGATTGGTAGAGA-3′, for *Actin*, forward 5′-CAACGAGCGGTTCCGAT-3′ and reverse 5′-GCCACAGGTTCCATACCCA-3′, *Tbp,* (Mm00446971_m1).

Real-time PCR was performed on the Step One Plus Detection System (Applied Biosystems) employing SYBR Green PCR Master Mix (Applied Biosystems). Each reaction mixture contained 7.5 μL of cDNA, whose concentration was 2 μg, 0.75 μL of each primer (whose concentration was 100 nM), and 7.5 μL of SYBR Green PCR Master Mix (2X) and for TaqMan gene expression assays (Applied Biosystems), each 20 μL of TaqMan reaction, 9 μL cDNA (18ng) was mixed with the 1 μL 20x probe of TaqMan Gene Expression Assays and 10 μL of 2X TaqMan Universal PCR Master Mix.

Data were analyzed utilizing the comparative Cycle threshold (Ct) method (ΔΔCt), where the actin transcript level was utilized to normalize differences in sample loading and preparation. Each sample (n=4(0002013)5) was analyzed in triplicate, and the results represented the n-fold difference of transcript levels among different samples.

### Statistical analysis

Data are expressed as the mean ± Standard Error of the Mean (SEM) from at least 4(0002013)5 samples. Data analysis was conducted using GraphPad Prism ver. 6 statistical software. Means were compared with two-way Analysis of Variance (ANOVA) and post hoc analysis. Comparisons between groups were performed by the unpaired Student *t* test for independent samples. Statistical significance was considered when *p* values were <0.05. Statistical outliers were performed out with the Grubs test and were removed from analysis.

In addition, partial correlation controlling for group were calculated using SPSS 19.00, between the following variables: DI, Time in open arms, Locomotor activity, Amyloid plaques, SOD1, 4-HNE, DNMT1 and HDAC2. Partial correlation coefficients were calculated using the data from number of Amyloid plaques obtained with S-Thioflavin staining; behavioral parameters from NORT, EPM and OFT; protein expression for SOD1 and 4-HNE; and gene expression for *Dnmt1* and *Hdac2*.
